# Genetic diversity and population divergence of *Leonurus japonicus* and its distribution dynamic changes from the last interglacial to the present in China

**DOI:** 10.1186/s12870-023-04284-x

**Published:** 2023-05-25

**Authors:** Yiheng Wang, Jingyi Wang, Thomas Avery Garran, Hangxiu Liu, Huaibin Lin, Jun Luo, Qingjun Yuan, Jiahui Sun, Wenpan Dong, Lanping Guo

**Affiliations:** 1 State Key Laboratory Breeding Base of Dao-di Herbs, National Resource Center for Chinese Materia Medica, China Academy of Chinese Medical Sciences, Beijing, 100700 China; 2 Key Laboratory of Biology and Cultivation of Herb Medicine, Ministry of Agriculture and Rural Affairs, Beijing, 100700 China; 3 Kunming Xishan Forestry and Grassland Comprehensive Service Center, Kunming, 650118 China; 4 Laboratory of Systematic Evolution and Biogeography of Woody Plants, School of Ecology and Nature Conservation, Beijing Forestry University, Beijing, 100083 China

**Keywords:** *Leonurus japonicus*, Plastid genetic diversity, Phylogenetic relationship, Population divergence, Distribution dynamic changes, Suitable climatic conditions

## Abstract

**Background:**

*Leonurus japonicus*, a significant medicinal plant known for its therapeutic effects on gynecological and cardiovascular diseases, has genetic diversity that forms the basis for germplasm preservation and utilization in medicine. Despite its economic value, limited research has focused on its genetic diversity and divergence.

**Results:**

The avg. nucleotide diversity of 59 accessions from China were 0.00029 and hotspot regions in *petN-psbM* and *rpl32-trnL*_*(UAG)*_ spacers, which can be used for genotype discrimination. These accessions divided into four clades with significant divergence. The four subclades, which split at approximately 7.36 Ma, were likely influenced by the Hengduan Mountains uplift and global temperature drop. The initial divergence gave rise to Clade D, with a crown age estimated at 4.27 Ma, followed by Clade C, with a crown age estimated at 3.39 Ma. The four clades were not showed a clear spatial distribution. Suitable climatic conditions for the species were identified, including warmest quarter precipitation 433.20 mm ~ 1,524.07 mm, driest month precipitation > 12.06 mm, and coldest month min temp > -4.34 °C. The high suitability distribution showed contraction in LIG to LGM, followed by expansion from LGM to present. The Hengduan Mountains acted as a glacial refuge for the species during climate changes.

**Conclusions:**

Our findings reflected a clear phylogenetic relationships and divergence within species *L. japonicus* and the identified hotspot regions could facilitate the genotype discrimination. The divergence time estimation and suitable area simulation revealed evolution dynamics of this species and may propose conservation suggestions and exploitation approaches in the future.

**Supplementary Information:**

The online version contains supplementary material available at 10.1186/s12870-023-04284-x.

## Background

*Leonurus japonicus* Houtt. is an annual or biennial herbaceous flowering plant that belongs to the Lamiaceae family. This species was first described in “Shennong Bencao jing,” a famous herbal work from the Han dynasty of China, and has been utilized in traditional Chinese medicine for over two thousand years. The center of distribution for *L. japonicus* is located in China, where it is commonly referred to as “Yi Mu Cao,” which translates to “beneficial to mothers.” This literal meaning implies its function: it has therapeutic effects in gynecological and cardiovascular disease [1]. Despite the long history of use for *L. japonicus* in traditional Chinese medicine, much of the existing research on this species has focused on its chemical constituents, pharmacological activity, and clinical applications, with comparatively little attention paid to the genetic diversity of its germplasm resources [1–5].

Genetic diversity is considered to be the amount of species’ genetic variability among individuals or populations [6]. Multidimensional differences between individuals or populations, such as biochemical characteristics, physiological properties, and morphological characters, are induced by genetic diversity. Moreover, for medicinal plants, the chemotypes of diverse genotypes’ germplasm resources are directly related to therapeutic efficacy [7]. It follows that genetic diversity is the basis of germplasm utilization and conservation, especially for medicinal plants. Previous studies have pointed out that in long-cultivated crops or medicinal plants, reduced genetic diversity caused by foundation effects or germplasm hybridization frequently results in germplasm degradation, and the level of degradation is correlated with the time of cultivation [8–12]. As an herbal medicine with a long cultivation history, *L. japonicus* may face the same situation; therefore, assessing its genetic diversity and population divergence are the first steps to determining the extent of its germplasm degradation. These steps are also prerequisites to deciding how to use, as well as how to conserve, the germplasm resources in order to stop or slow down the process of germplasm degradation. Recently, most germplasm protection or utilization measures have mainly been intuitively based on morphological or chemical traits, moving forward with little information on the genetic diversity and population divergence. However, this situation needs to be rectified.

Understanding the habitat and climate dynamics of species is also indispensable, since the genetic diversity of species is significantly shaped by geographic changes and climate oscillations [13]. Dispersal of species in response to climate oscillations, especially during glacial and interglacial periods, accelerates species/population divergence [14]. Furthermore, adaptive genetic variation has been shown to be closely related to climate factors [15–18]. Therefore, to propose effective measures for the conservation and utilization of *L. japonicus*, it is essential to integrate genetics and climate data.

Owing to the development of next-generation sequencing technologies, [19]the high cost of extensive sequencing is significantly reduced, which facilitates the evaluation of genetic diversity and population divergence [20]. Due to their stable structure, rare recombination, and moderate evolution rate, plastomes have been widely used in genetic diversity and population divergence estimation, as well as in different germplasm discrimination of medicinal plants [19, 21]. Additionally, in order to reveal the impact of climate oscillations on species, the maximum entropy (MaxEnt) model based on niche theory is highly favored and has been widely used to reveal the connection between climate and species distribution, and to simulate suitable distribution dynamics for species [22–24].

In this study, we conducted nationwide *L. japonicus* sampling work, newly sequencing and assembling plastomes of 59 *L. japonicus* accessions from almost all its habitats in China. Combined with the geographical and climatic data from the Last Interglacial (LIG) to the present in China, we aimed to (1) evaluate the genetic diversity and population divergence of *L. japonicus*, (2) elucidate its evolutionary history and (3) simulate its suitable distribution dynamics from past to present. We are attempting to propose long-term conservation strategies and more reasonable exploitation approaches for this historic medicinal plant by merging the results of these aspects.

## Results

### Plastome features and phylogeny reconstruction

Plastomes of 59 *L. japonicus* accessions were obtained using a genome skimming approach and deposited in GenBank with the accession numbers listed in Table S1. The total plastome length of accessions ranged from 151,395 bp (accession YMC62) to 151,733 bp (accession YMC74) in length and was composed of a typical quadripartite structure, including a pair of IR regions (25,625–25,646 bp), LSC regions (82,499–82,839 bp), and SSC regions (17,499–17,640 bp). The average GC content was 38.4% in total, with no significant difference among *L. japonicus* accessions. The plastomes possessed 113 unique genes, including 80 protein-coding genes, 29 transfer RNA genes, and four ribosomal RNA genes.

**Fig. 1:**
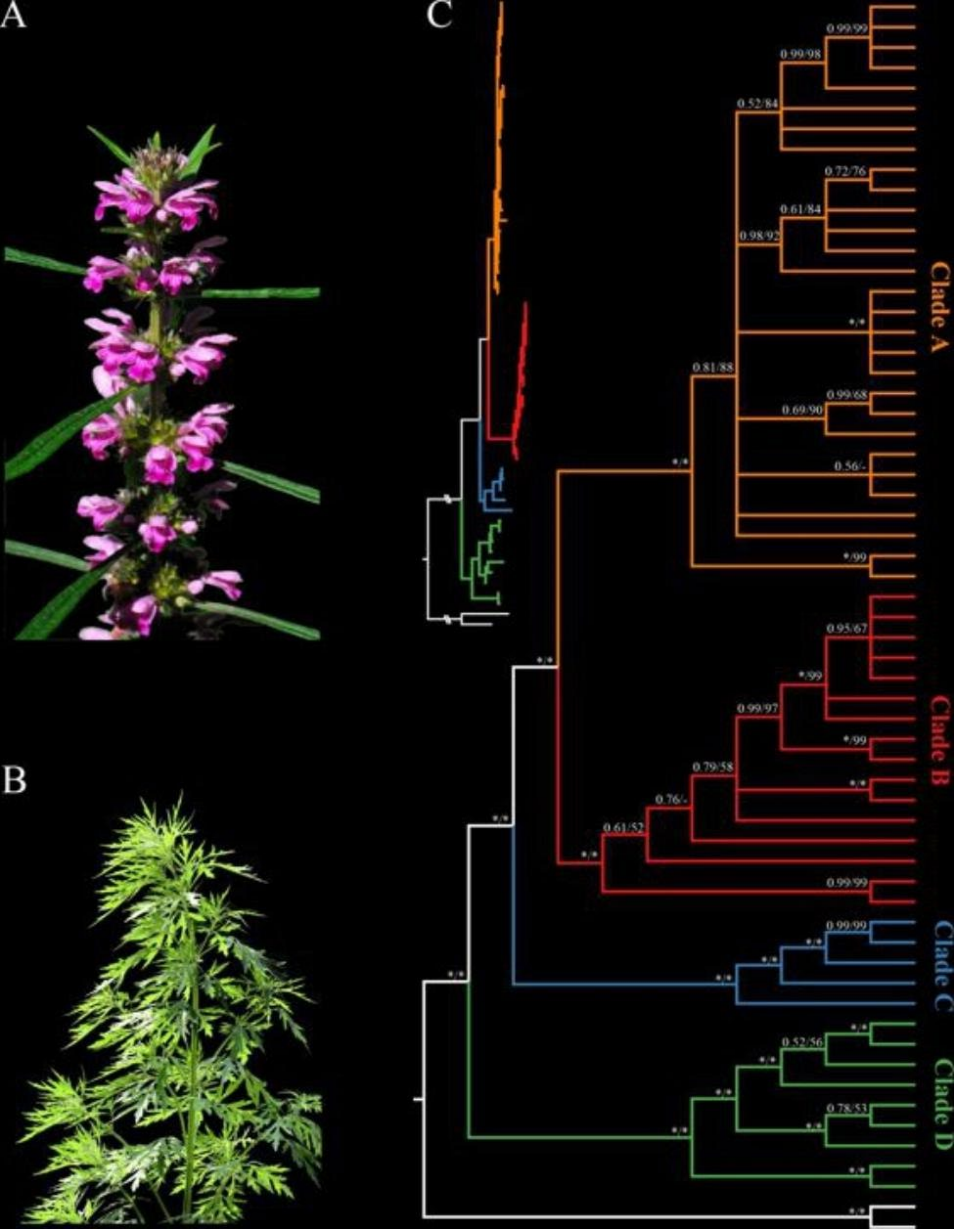
Morphological characters of *L. japonicus* flowers (**A**) and seedlings (**B**); The phylogenetic analysis was conducted using maximum likelihood (ML) and Bayesian inference (BI) of 59 *L. japonicus* accessions (**C**). Numbers on branches correspond to Bayesian posterior probability (PP) and ML bootstrap support (BS), respectively. An asterisk (*) indicates BS = 100% or PP = 1.0.

We used ML and BI methods for phylogeny reconstruction based on entire genome sequences, taking two *L. cardiaca* accessions as outgroups. The topologies and cluster of the accessions calculated by the two algorithms were almost consistent (Fig. 1). The results of the phylogeny suggested that the 59 *L. japonicus* accessions from all over its distribution area in China should be clustered into four subclades, namely Clades A to D, marked with different colors in Fig. 1.

### Plastome diversity and hotspot identification

**Fig. 2:**
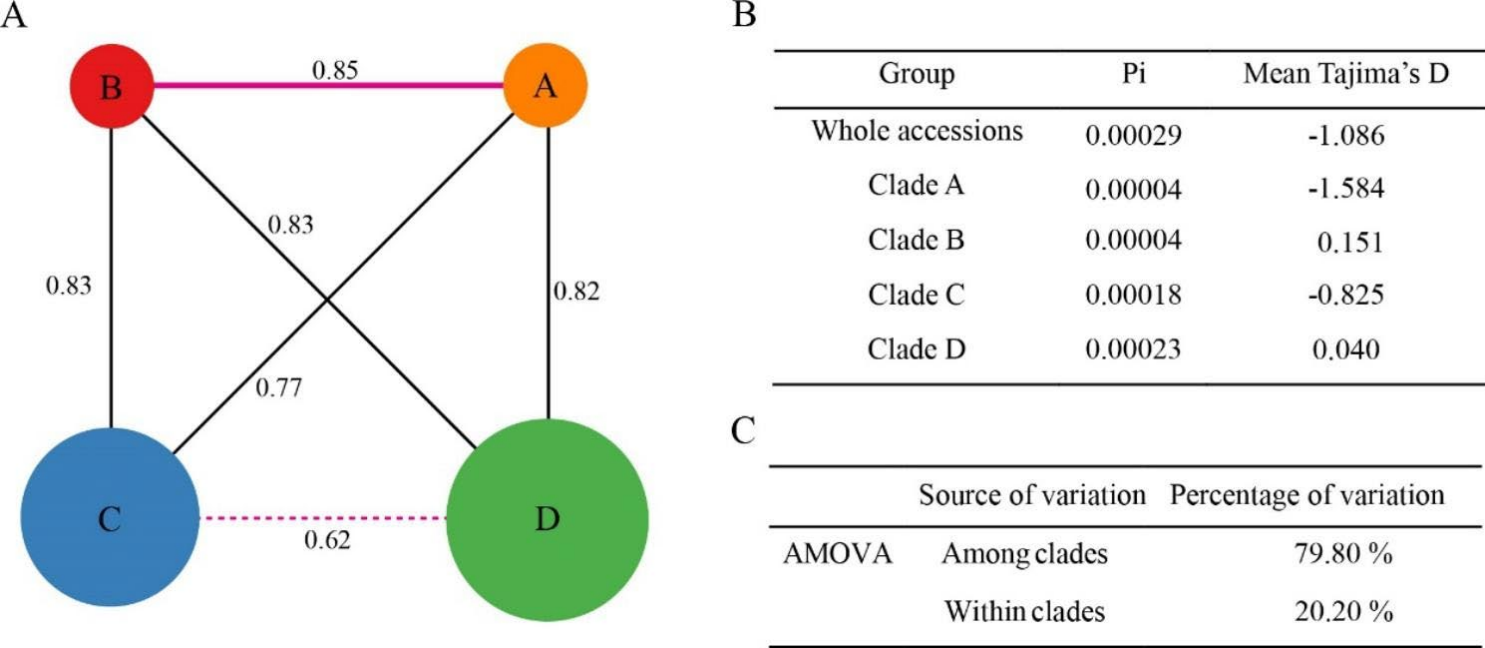
Plastome diversity of 59 *L. japonicus* accessions. (**A**) the Fst among four subclades (circles in different colors); the Fst values are listed on lines, the circle sizes represent the Pi of different subclades. (**B**) Average Pi and Tajima’s *D* for the entire collection and each subclade. (**C**) Analyses of molecular variance (AMOVA) of accessions

The alignment of 59 *L. japonicus* accessions was 151,980 bp in length. We recovered 291 variable sites, 49 haplotypes, and the average Pi of the total accessions was 0.00029. The average Pi of Clades A to D was 0.00004, 0.00004, 0.00018, and 0.00023, respectively. Clade D contained the highest nucleotide diversity among the four clades, while Clades A and B contained the lowest (Fig. 2B). We also carried out the selection analysis via Tajima’s *D* statistic; high values of Tajima’s *D* indicate an excess of common variation, which may be consistent with balancing selection or population contraction, whereas negative values indicate an excess of rare variation, consistent with increasing population size or positive selection. The results of Tajima’s *D* showed that the *L. japonicus* in China, as a whole, suffered from positive selection pressure. Specifically, Clade A and Clade C were under positive selection or population increases, Clade B was under balancing selection or a population decrease, and rare selective pressure acted on Clade D. The Fst values ranged from 0.62 to 0.85, presented on the lines in Fig. 2A, indicating that the subclades of *L. japonicus* in China were highly differentiated. Analysis of molecular variance (AMOVA) revealed similar conclusions, indicating that most of the genetic diversity existed among subclades (79.80%, P < 0.001) (Fig. 2C).

**Fig. 3:**
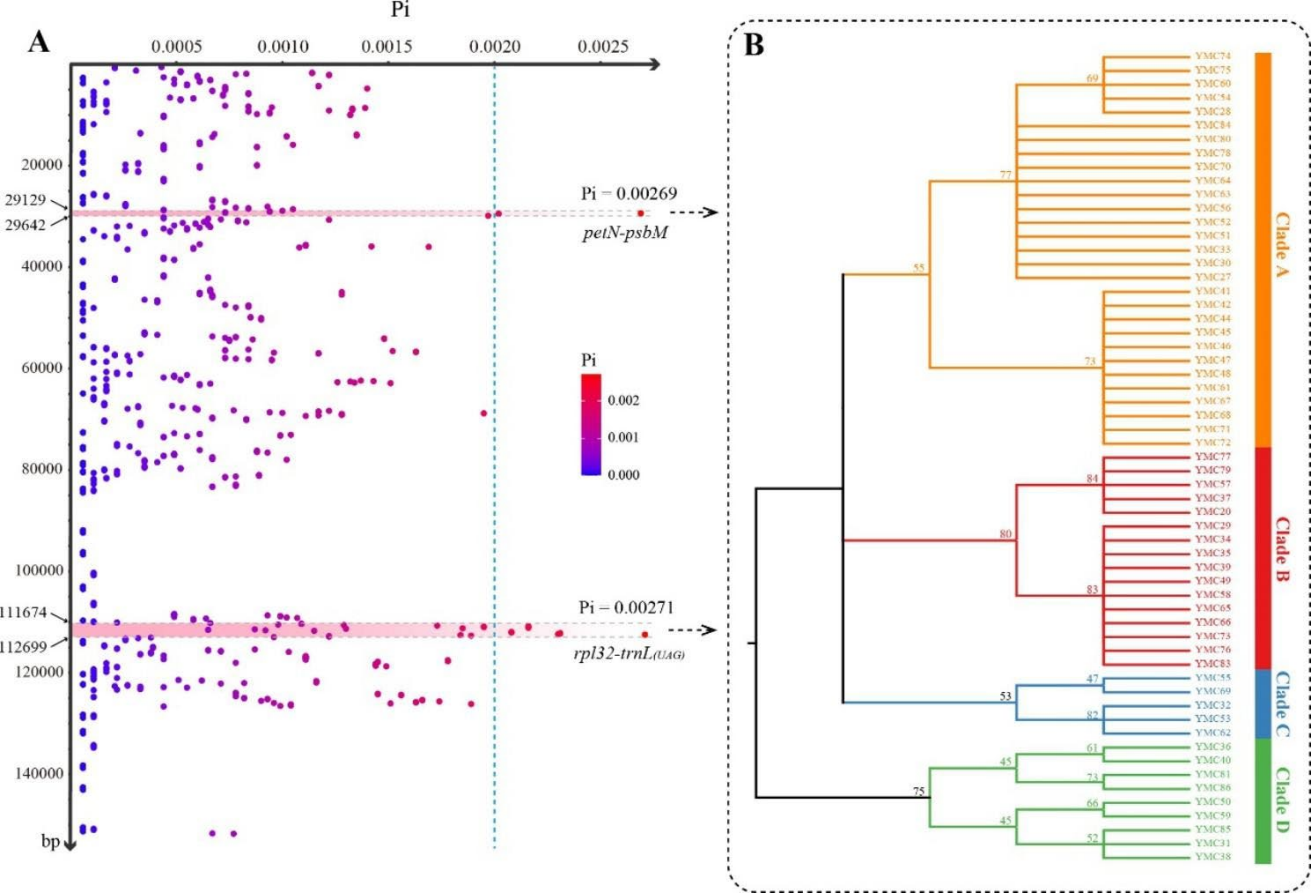
Hotspot identification of 59 *L. japonicus* accessions. (**A**) Sliding-window analysis of the plastomes of 59 *L. japonicus* accessions. The horizontal coordinate represents Pi value and the vertical coordinate represents the window location in the plastome. (**B**) A neighbor-joining (NJ) tree built by combining two hotspot regions (*petN–psbM* and *rpl32–trnL*_*(UAG)*_).

The average Pi of 600 bp sliding windows varied from 0 to 0.00271. We selected the top 1% of the windows as hotspot regions, which were mainly concentrated in two spacer regions of *petN–psbM*, 513 bp in size, and *rpl32–trnL*_*(UAG)*_, 1025 bp in size (Fig. 3A). When the two hotspot regions were combined to build a neighbor-joining (NJ) tree, the topology of the tree clustered into four subclades with a relatively high support value, which was identical to the structure of the whole-plastome sequences (Fig. 3B).

### Genetic structure of ***L. japonicus*** subclades

**Fig. 4:**
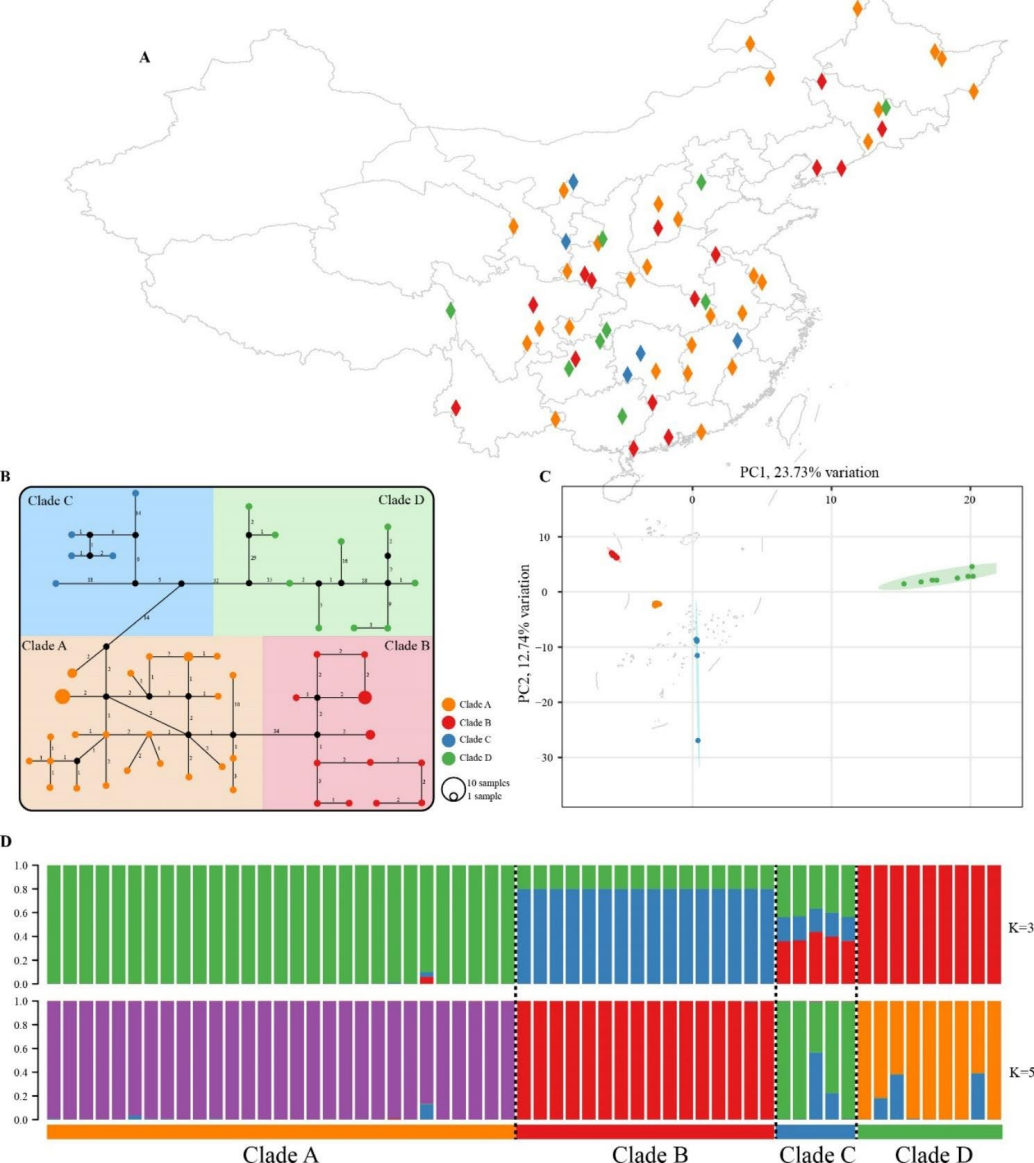
Population structure of *L. japonicus* accessions. (**A**) Geographic distribution of the sampling locations. Different colors represent different subclades. The map plot was generated using ArcGis 10.8. (**B**) TCS network for all 59 accessions. Missing haplotypes are indicated by black dots and mutational steps are indicated by numbers. (**C**) PCA of *L. japonicus* accessions; the proportion of the variance explained was 23.73% for PC1 and 12.74% for PC2. (**D**) STRUCTURE analysis for K = 3 and 5. Colors indicate different clusters. The x-axis shows the subclades, and the y-axis indicates the probability of inferred ancestral lineages

To visualize the genetic structure of *L. japonicus* subclades, a comprehensive analysis was conducted using 291 single nucleotide polymorphisms (SNPs) obtained from the 59 accessions. This analysis employed a combination of STRUCTURE analysis, principal component analysis (PCA), and network analysis. The results of the PCA revealed a marked separation of accessions, which clustered into four distinct subclades (Fig. 4C). The most suitable “blood lineages” of *L. japonicus* in China were determined using the DeltaK, and the largest value was K = 5, followed by K = 3 (Figure S2). According to the two suitable K-values, the structure result of the 59 accessions is presented in Fig. 4D, showing that the accessions could be clearly distinguished into four groups, and that little gene flow has occurred between groups. The results of the TCS network analysis, based on 49 haplotypes, also supported the clustering of the accessions into four distinct groups (Fig. 4B). These findings, obtained from three different analytical methods, all led to the same conclusion of significant divergence between the clades and rare gene flow. Interestingly, when the sample locations were plotted on a map of China, it was observed that the genetic clustering of the accessions was not correlated with their geographical distribution, suggesting that the germplasm of *L. japonicus* was highly mixed (Fig. 4A).

### Divergence time estimation of ***L. japonicus*** clades

**Fig. 5:**
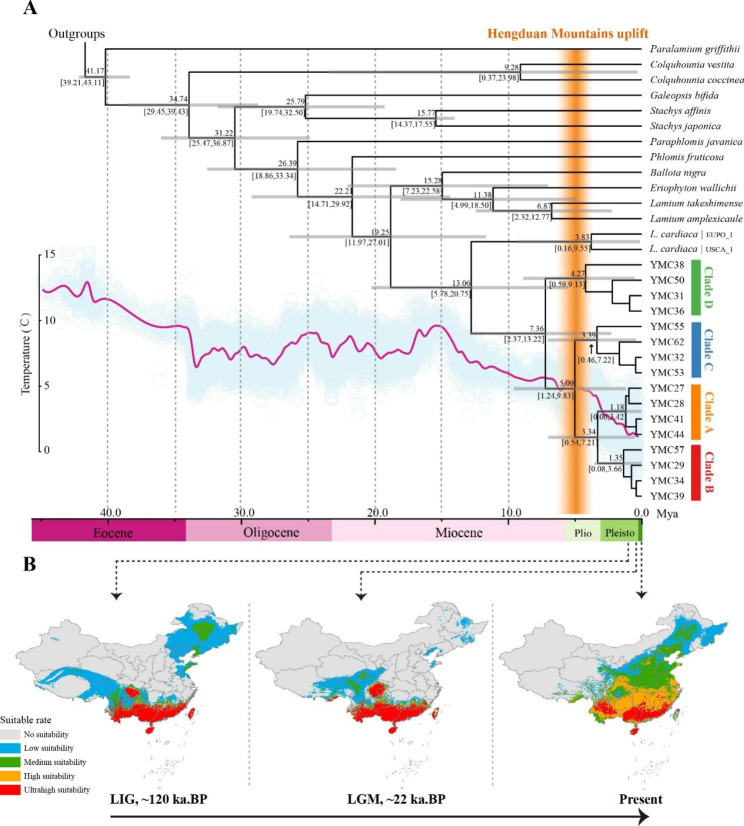
Combined dating analyses and suitable distribution dynamic changes of *L. japonicus*. (**A**) Numbers above and under the branches of the dated phylogeny indicate the mean divergence times and 95% confidence interval of each node, respectively. Gray bars indicate the 95% highest posterior density intervals. The global temperature change in the past 45 Ma was obtained from Zachos et al. [25]. The time of the Hengduan Mountains uplift is shown in an orange histogram. (**B**) Suitable distribution simulation of *L. japonicus* in LIG, LGM, and the present. Five levels of suitability are shown in different colors as follows: no suitability (0–0.2, gray); low suitability (0.2–0.4, blue); medium suitability (0.4–0.6, green); high suitability (0.6–0.8, yellow) and ultrahigh suitability (0.8–1, red)

According to the divergence time estimation, the divergence of *L. japonicus* and *L. cardiaca* from their last common ancestor occurred around 19.25 Ma, during the early Miocene, with a subsequent split from each other in the mid-Miocene, at approximately 13.06 Ma (Fig. 5). An analysis of the phylogenetic relationships within *L. japonicus* reveals a series of divergence events, beginning with the first split occurring approximately 7.36 Ma. This initial divergence gave rise to Clade D, with a crown age estimated at 4.27 Ma, followed by Clade C, with a crown age estimated at 3.39 Ma. Finally, the divergence between Clades A and B occurred at around 3.34 Ma, with crown ages estimated at 1.18 Ma and 1.35 Ma, respectively. Furthermore, it has been observed that the diversification of the different subclades within *L. japonicus* was largely influenced by the uplift of the Hengduan Mountains and coincided with a global temperature drop.

### Dominant climatic variables and habitat dynamic changes

**Fig. 6:**
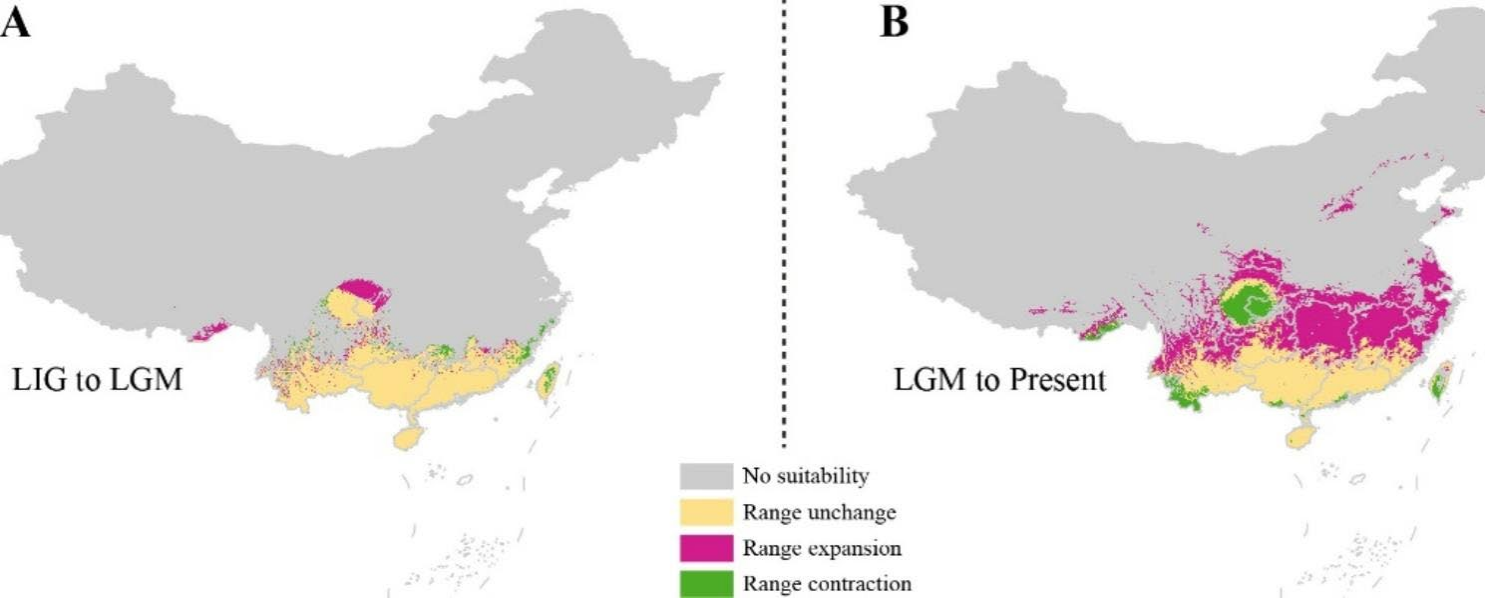
The shifts of distribution areas between adjacent time periods under suitability conditions > 0.6. (**A**) LIG to LGM. (**B**) LGM to the present

The MaxEnt modeling of *L. japonicus* had good accuracy and robustness for prediction: the average test AUC was 0.82, with a standard deviation of 0.029. The nine selected climatic variables involved in the habitat simulation and their contribution weights are listed in Table S3. Precipitation of the warmest quarter (Bio 18) was the variable with the highest contribution rate (53.7%), followed by precipitation of the driest month (Bio 14, contribution rate 17.2%) and the minimum temperature of the coldest month (Bio 6, contribution rate 13.1%). Overall, precipitation was seemed given more weight. These three variables were the dominant climatic factors. According to the response curve of the three dominant climatic variables based on the high suitability criterion of 0.6, the optimum ranges of precipitation of the warmest quarter should lie within the range of 433.20 to 1524.07 mm, the precipitation of the driest month should exceed 12.06 mm, and the minimum temperature of the coldest month should exceed − 4.3 °C (Figure S3). Collectively, precipitation had a greater impact on *L. japonicus* suitability. The current ecological habitat for *L. japonicus* predicted by MaxEnt modeling extensively matched its present distributions, and the simulated area of the four suitable levels (suitability rate > 0.2) from high to low were 49.7 × 10^4^, 122.3 × 10^4^, 149.5 × 10^4^, and 125.7 × 10^4^ km^2^, respectively (Fig. 5B and S4). The paleodistribution ranges showed a dynamic change of contraction (LIG to LGM) followed by expansion (LGM to present), mainly caused by the large temperature fluctuations during glacial periods. In particular, the areas with a suitability rate > 0.6 (including high and ultrahigh suitability) in LIG and LGM were mostly concentrated close to and south of the Tropic of Cancer, and in the Hengduan Mountains. Interestingly, the ultrahigh suitability area (suitability rate > 0.8) in the Hengduan Mountains was greatly increased during the LIG–LGM period, followed by dramatic shrinking (LGM–present). Overall, the distribution center from LIG to the present shows a northeastward shift from southwest to east-central China. In terms of areas with a suitability rate > 0.6, there has a pronounced increase from the LIG to the present (Fig. 6).

## Discussion

### Genetic diversity of ***L. japonicus*** in China

The practice of introducing and cultivating medicinal plants has long been associated with human intervention, which inevitably results in alterations to the genetic diversity of these species. The genetic diversity of a species plays a crucial role in determining its potential for evolution and resilience, and serves as the foundation for the preservation and utilization of germplasm resources in the field of medicinal plant research [26]. As such, it is imperative that the genetic diversity of *L. japonicus* be thoroughly understood. However, there is currently no comprehensive plastid genetic resource available for *L. japonicus*.

In this study, the genetic diversity of *L. japonicus* (Pi = 0.00029) was found to be similar to that of its close relative, *L. cardiaca*, with an average Pi of 0.00042. Interestingly, a relatively low level of genetic diversity seems common among medicinal plants with a long history of cultivation and use, such as *Coptis chinensis*, *Ziziphus jujuba*, *Angelica sinensis*, and *Panax ginseng* [11, 27–29]. This may be the result of genetic bottlenecks caused by human activity, such as the cultivation of the species for personal medicinal use, which inevitably leads to the reduction of genetic diversity. To mitigate the exacerbation of germplasm degradation, measures should be taken to suppress the intensification of genetic bottlenecks of *L. japonicus*. The genetic structure of this species provides ideas for measures to be taken; the phylogeny and population structure results in Figs. 1 and 4 and S5 clearly indicate that *L. japonicus* in China can be divided into four clades with significant divergence between each other and rare gene flow, and we can treat these clades as four genotypes. Compared to other clades, accessions within Clade D diverged earlier and had a longer evolutionary time, which may account for the highest genetic diversity of Clade D and the second highest genetic diversity of Clade C.

### Genetic clusters and geographical distributions

Interestingly, the genetic clusters were not correlated with their geographical distributions. As a medicinal plant, local resources of *L. japonicus* were initially employed by the inhabitants. If it remained in this stage, it seemed likely that there would be some correlation between genetic clustering and geographic distribution. However, with the widespread commercial production of medicinal plants, a considerable number of seedlings have been frequently exchanged between different regions. Subsequently, these seeds have spread beyond the plantation and escape into the wild. Notably, these escaped accessions exhibit a greater genetic affinity with their origins. While it appears that geographically these accessions disrupt the existing differentiation pattern among the original populations, it is challenging to trace their initial origin given their recent occurrence. Overall, the findings of this study firmly establish that despite the frequent trading of seedlings, *L. japonicus* in China originates from four fundamental sources.

### Evolutionary history and distribution dynamic changes

In this study, we conducted a comprehensive and meticulously resolved phylogenetic analysis of *L. japonicus* and its related species. The results showed that the accessions of *L. japonicus* form a monophyletic group and, within the species, divergence events took place between 7.36 Ma during the late Miocene period, leading to the formation of four subclades. This period of diversification coincided with the rapid uplift of the Hengduan Mountains after the Miocene and reached its peak before the Late Pliocene [30]. In fact, Himalaya–Hengduan Mountains is a well-known biodiversity center, and is referred to as “the largest evolutionary front of the North Temperate Zone” [31]. Continents were covered in ice as global temperatures dropped, but the unique geographical location and climatic conditions of the Hengduan Mountains region made it a refuge for many plants [32, 33]. In the southern area of the Tropic of Cancer, the precipitation levels remained relatively stable, and the climate remained humid since the Last Glacial Maximum, as revealed by hydrogen and oxygen isotopes [34]. This is reflected in our simulated paleodistribution ranges of LIG and LGM, which showed that *L. japonicus* was most likely distributed in close proximity to and south of the Tropic of Cancer, and in the Hengduan Mountains. The refuge effect was strengthened in the Hengduan Mountains as the temperature dropped, leading to an expanded distribution range in the LGM. As the climate stabilized, the species spread to other regions in China. Overall, the results of the phylogeny and suitable distribution dynamics of *L. japonicus* highlight the complex and dynamic history of this species and the importance of continued study to gain a deeper understanding of its evolutionary history and genetic structure.

### Conservation and utilization of ***L. japonicus***

The conservation and utilization of medicinal plant species like *L. japonicus* is heavily reliant on a better understanding of its genetic diversity. This understanding is critical for making informed decisions about what to conserve and how to use the species [6]. From the preceding results, it can be seen that *L. japonicus* in China faces two problems: genetic bottlenecks and germplasm mixing. Clarifying the genetic background (genotypes) is a prerequisite for solving these two problems. Although the cost of plastome acquisition has largely decreased with the development of next-generation sequencing, it is still cost-prohibitive work if applied to large sample sizes. The hotspot regions (two spacer regions: *petN–psbM* and *rpl32–trnL*) identified by sliding window analysis in *L. japonicus* plastomes will help us clarify different genotypes in a more cost-effective way, and could be accessed through two polymerase chain reactions (PCR). Moreover, these two markers were capable of forming accessions into four subclades with high support value. The sample composition of each subclade was identical to whole-plastome sequences, which indicated that these two fragments could be taken as efficient markers for *L. japonicus* genotype discrimination. These two fragments have also been used in phylogenetic analyses of *Cistus*, *Bambusa*, and *Juniperus* [35–37]. Clarify the genotype background could help to alleviate the exacerbation of genetic bottlenecks, and protect the species in the long term. A germplasm seed bank is an effective way for conservation. A genotype-guided approach is more rational and valuable for establishing a germplasm seed bank and promoting a core germplasm collection while reducing disorderly seed exchange of *L. japonicus*. This study has comprehensively demonstrated the adverse ramifications of unregulated seedling exchange. From the standpoint of long-term species protection, the government should also be aware of this as soon as possible and take suitable measures.

Additionally, the suitable conditions for *L. japonicus* in terms of precipitation and temperature (precipitation of the warmest quarter from 433.20 to 1524.07 mm, precipitation of the driest month > 12.06 mm, and the minimum temperature of the coldest month > -4.34 °C) can contribute to the selection of germplasm nursery sites and will also be useful for guiding cultivation and introduction. Moreover, the genotype and chemotype of a medicinal plant are undoubtedly closely related, and the chemical composition of the medicinal plant directly affects its clinical effectiveness [7]. Therefore, the next step is to clarify the content of therapeutically relevant components corresponding to different genotypes of *L. japonicus* for better utilization.

## Conclusions

In this study, the phylogeny suggested that the *L. japonicus* in China should be clustered into four subclades represented four genotypes. Comparative analysis revealed that accessions from these four genotypes were highly differentiated and the identified hotspot regions could be used for genotype discrimination. Within the species, divergence events started during the late Miocene, and may be related to the rapid uplift of the Hengduan Mountains. In Quaternary glaciation, the Hengduan Mountains played the role of a refuge for *L. japonicus.* By MaxEnt modeling, we visualized the habitat dynamic changes and clarified the optimal precipitation and temperature range for *L. japonicus*. Overall, our findings provide insights into the genetic diversity, divergence, and evolution of *L. japonicus* and suggests suitable conservation and exploitation approaches.

## Methods

### Plant material, DNA extraction, and occurrence records

59 *L. japonicus* accessions were collected from 24 provinces in China during the Fourth National Survey of Chinese Materia Medica Resources, and only four of which were cultivated accessions. The voucher specimens were identified by Jiahui Sun and deposited at the herbarium of CMMI (Institute of Chinese Materia Medica, China Academy of Chinese Medical Sciences, Beijing, China) with deposition number listed in Table S1. Total DNA was extracted using a modified cetyl trimethyl ammonium bromide (mCTAB) method [38]. The occurrence records of *L. japonicus* in China were collected from the Global Biodiversity Information Facility (GBIF; occurrence download 10.15468/dl.zqzb7b) and the National Plant Specimen Resource Center (NSII; http://www.nsii.org.cn/). After removing or correcting synonyms, unresolved names (checked by The Plant List, http://www.theplantlist.org), and invalid distribution points (checked by ArcGis 10.8), 417 distribution records of *L. japonicus* were obtained for further analysis.

### Plastome sequencing, assembly, and annotation

After fragmenting the extracted DNA into 300–350 bp by sonication, a pair-end library was constructed using the NEBNext Ultra™ DNA library prep kit (New England Biolabs, Ipswich, MA, USA). PE150 sequencing was performed on the Illumina HiSeq XTen platform at Novogene Co., Ltd (Beijing, China). The clean data were generated from the PE150 raw data by using Trimmomatic 0.39 software for quality control and filtering (with settings: ILLUMINACLIP:TruSeq3-PE. fa:2:30:10:1:true LEADING:20 TRAILING:20 SLIDINGWINDOW:4:15) [39]. Getorganelle v1.7.5 software was used for *de novo* assembly with settings: -R 15 and -k 105 [40]. Plastome annotation was performed using program CPGAVAS2, taking “2544 plastomes” as a reference dataset and manually checking for missing or incorrectly annotated genes in Sequin [41].

### Population structure and genetic relationships

A total of 59 plastomes were aligned using MAFFT online service in auto strategy [42], and manually adjusted by Se-al 2.0 [43]. Haplotype data were analyzed in DnaSP v5.10, and Arlequin v3.5.1.3 was used to determine the haplotype frequencies in populations [44]. A TCS network of 59 plastomes was generated with PopArt v1.7 using the haplotype data and population haplotype frequencies data [45, 46].

A matrix containing 291 SNPs was generated in DnaSP v5.10, and sites with alignment gaps were excluded [47]. The SNPs matrix was converted into a suitable format using GenAlEx v6.5 and used for STRUCTURE and PCA [48]. The R package “PCAtools” was used to preform PCA and make PCA plots. The STRUCTURE workflow was according to the previous study [11]: (1) The K-means clustering algorithm was ran from K = 2 to K = 10 with ten runs for each K-value to determine the optimum number of clusters [49], (2) the initial burn-in period was set to 10,000 with 100,000 MCMCs, (3) the most suitable clusters were determined using the DeltaK method on Structure Harvester (http://taylor0.biology.ucla.edu/struct_harvest/) and aggregated by running the CLUMPP program [50], and (4) visualizing the structure plots by using R package “ggplot2” [51].

### Phylogenetic inference and genetic diversity in ***L. japonicus***

A total of 61 plastomes were used for phylogenetic inference, including two sequences of *Leonurus cardiaca* species from GenBank taken as outgroups (MZ274168 and MZ274153). The program ModelFinder was employed to find the best-fit model based on the Bayesian information criterion [52]. Two methods (maximum likelihood, ML, and Bayesian inference, BI) were carried out for phylogeny reconstruction. The ML tree was generated using IQ-TREE with the TVM + F + R4 model in PhyloSuite and nodes with bootstrap values below 50 were collapsed by TreeCollapseCL 4 (http://emmahodcroft.com/TreeCollapseCL.html) [53, 54]. The BI tree was inferred using MrBayes 3.2.6 [55] under a GTR + I + F model (12 parallel runs, 500,000 generations), in which the initial 25% of sampled data were discarded as burn-in. Trees were displayed in FigTree v1.3.1. The nucleotide diversity (Pi) of total accessions was calculated based on sliding window analysis using DnaSP v5.10 software with parameter settings of a 600-bp window length and 100-bp step length, and the point plot was visualized using ggplot2. The neighbor-joining (NJ) tree of hotspots was generated in MEGA 7. Detailed information about Pi, and Tajima’s *D* of *L. japonicus* subclades were also calculated using DnaSP v5.10. AMOVA and pairwise Fst calculations were performed in Arlequin v3.5 with default settings to assess the degree of divergence among subclades [44].

### Divergence time estimation

We selected four accessions from each subclade as representatives to create a dataset with other plastomes from Nepetoideae and Lamioideae for divergence time estimation (Table S2). The dataset was trimmed by Gblock before the dating analysis. A relaxed log normal clock model in the BEAST v2.6.6 platform was performed, using a GTR substitution model and a speciation Yule Process tree prior [56]. A fossil and two secondary calibration points were used for this analysis: (1) the *Stachys* clade was constrained at 13.8 Ma based on the oldest reliable lamioid fossils discovered from the Serravallian Age of the Middle Miocene flora of Germany [57], (2) the crown ages of Lamioideae and Mentheae were 41 Ma and 45.8 Ma, respectively, according to Rose et al. [58]. We ran the Markov Chain Monte Carlo (MCMC) chains for 500,000,000 generations and sampled every 10,000 generations. The effective sample size (ESS) was checked using Tracer v1.6 to ensure parameters exceeded 200. TreeAnnotator v2.6.6 was used to produce a maximum clade credibility (MCC) tree after discarding the first 25% as burn-in (http://beast.bio.ed.ac.uk/Tracer). Divergence time with 95% HPD intervals was displayed using FigTree v1.3.1 and modified in AI CS6.

### Climatic variables and distribution modeling

We performed spatial rarefaction of the obtained occurrence records at a 10 km resolution using SDM toolbox v2.5, and 259 records were left for MaxEnt modeling to reduce sampling deviation in simulating suitable distribution dynamics [59]. The environmental layers comprised 19 bioclimatic variables of three historic periods: the Last Interglacial (LIG; ~130 ka BP, spatial resolution in 30′′), the Last Glacial Maximum (LGM, ~ 22 ka BP, spatial resolution in 2.5′), and the present (1970–2000, spatial resolution in 2.5′) were downloaded from WorldClim (https://www.worldclim.org/) and applied for distribution prediction. All the climatic layers were converted to ASCII format using ArcGIS 10.8. To assure the accuracy and minimize overfitting, a Pearson correlation analysis of the 19 climate variables was conducted using the R package “ggpairs.” After removing the correlation pairs with |*r*| > 0.7, nine climate variables remained (Table S3 and Figure S1).

The MaxEnt modeling was carried out by setting ten cross-validation replicate runs, with 75% record sites used for model training and the remainder for validation. The modeling accuracy and robustness were evaluated by the area under the curve (AUC) value, which ranged from 0 to 1, with the following evaluation criteria: poor (0.6–0.7), fair (0.7–0.8), good (0.8–0.9), and excellent (0.9–1) [22]. The contribution rate and jackknife test were used to quantify the contribution weights of climatic variables. Results of suitable distribution modeling had a range of values from 0 to 1, which were regrouped into five grades using the equal interval approach: no suitability (0–0.2); low suitability (0.2–0.4); medium suitability (0.4–0.6); high suitability (0.6–0.8), and ultrahigh suitability (0.8–1). Additionally, the distribution changes between neighboring time periods were computed by SDM toolbox v2.5.

## Electronic supplementary material

Below is the link to the electronic supplementary material.

Supplementary Material 1

Supplementary Material 2

Supplementary Material 3

Supplementary Material 4

Supplementary Material 5

Supplementary Material 6

Supplementary Material 7

Supplementary Material 8

Supplementary Material 9

Supplementary Material 10

## Data Availability

59 plastomes generated in this study have been submitted to NCBI (https://www.ncbi.nlm.nih.gov) with accession numbers: OQ417534-OQ417592. The alignment of these 59 sequences has been uploaded in the Science Data Bank (https://www.scidb.cn/) with DOI:10.57760/sciencedb.07474.
