# Supplementary figures and images for: Genetic diversity and population divergence of Leonurus japonicus and its distribution dynamic changes from the last interglacial to the present in China
Source: BMC Plant Biol. 2023 May 25;23:276. doi: 10.1186/s12870-023-04284-x (PMC10210291; doi:10.1186/s12870-023-04284-x)

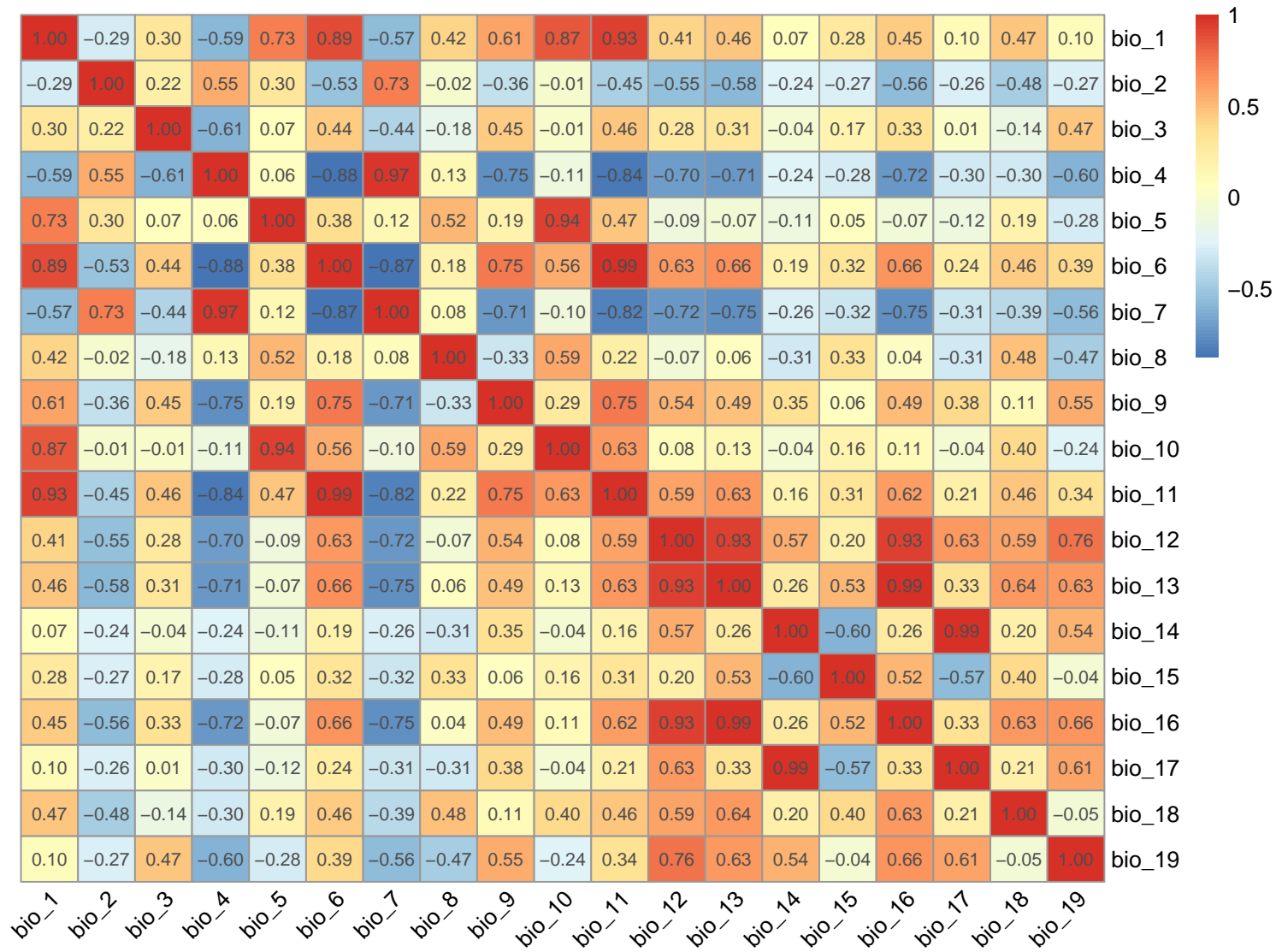

Supplement: Supplementary file 1 — Supplementary Material 1 [file 12870_2023_4284_MOESM1_ESM.pdf]

$$\text{DeltaK} = \text{mean}(|L''(K)|) / \text{sd}(L(K))$$

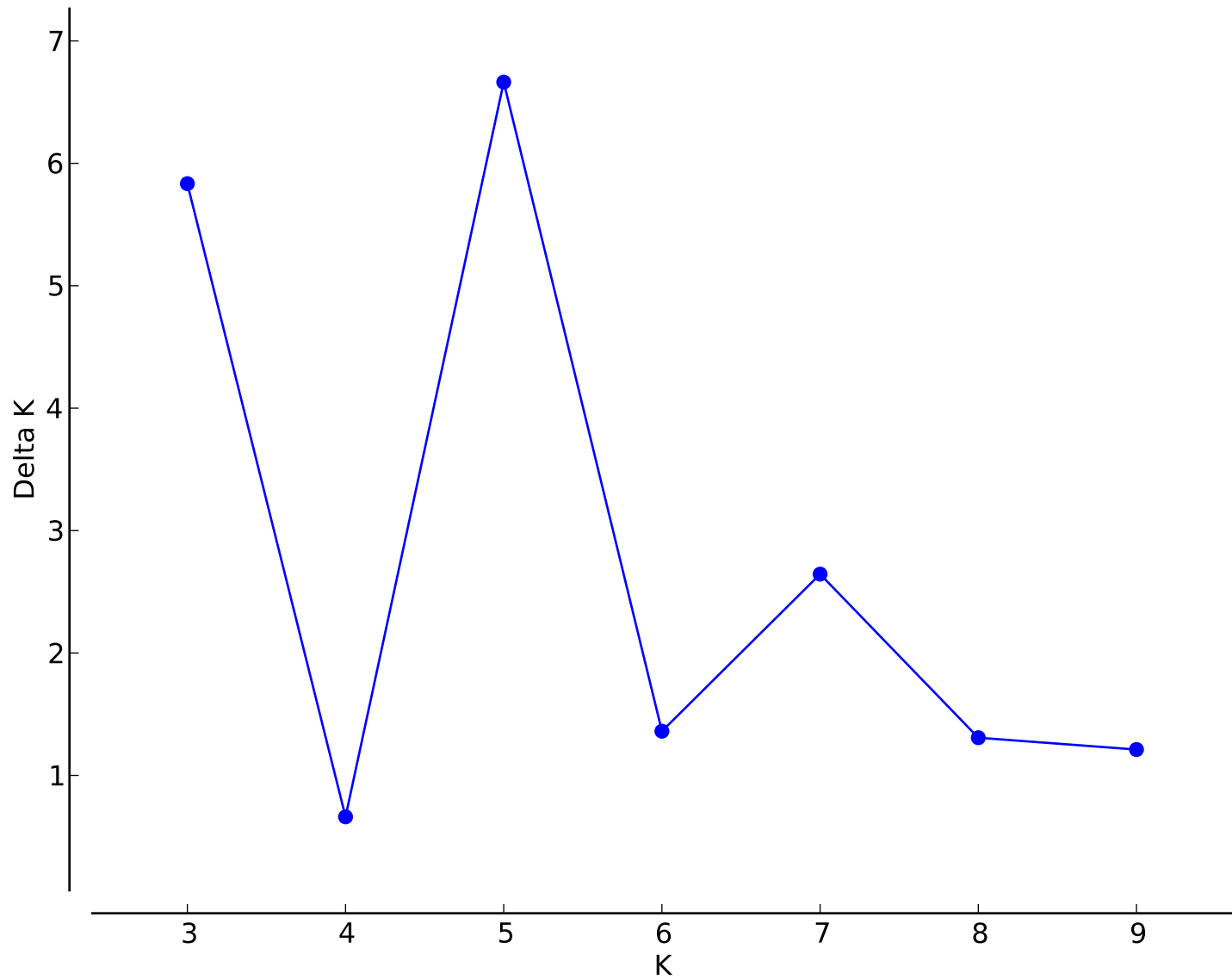

Supplement: Supplementary file 2 — Supplementary Material 2 [file 12870_2023_4284_MOESM2_ESM.pdf]

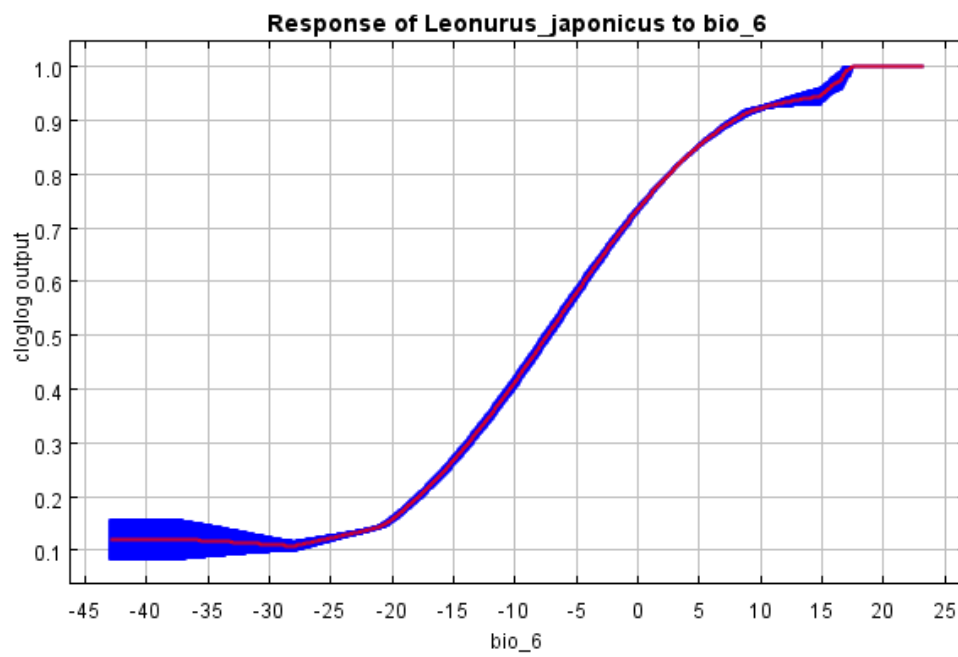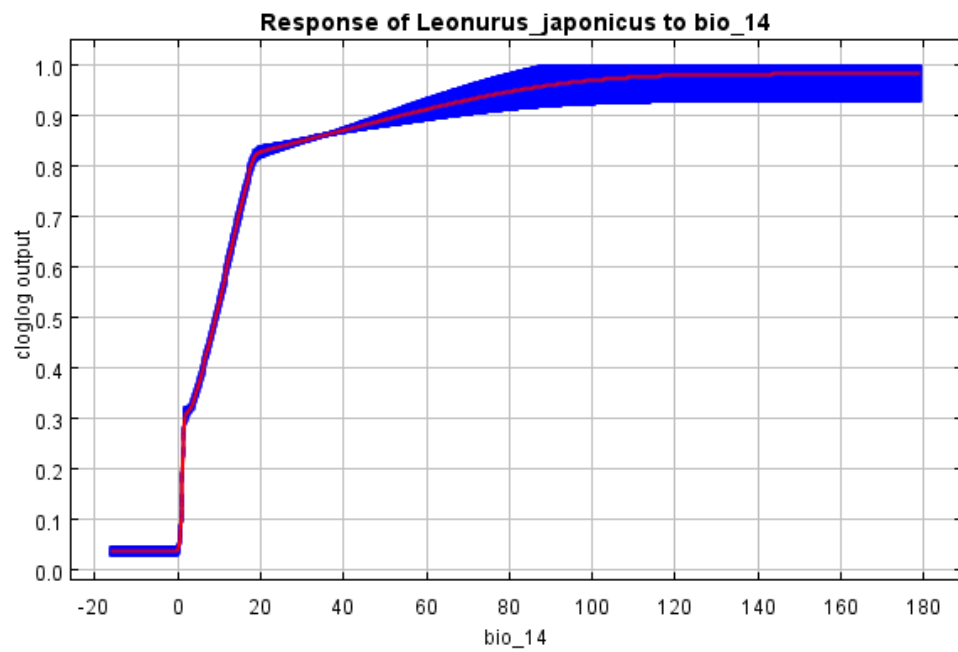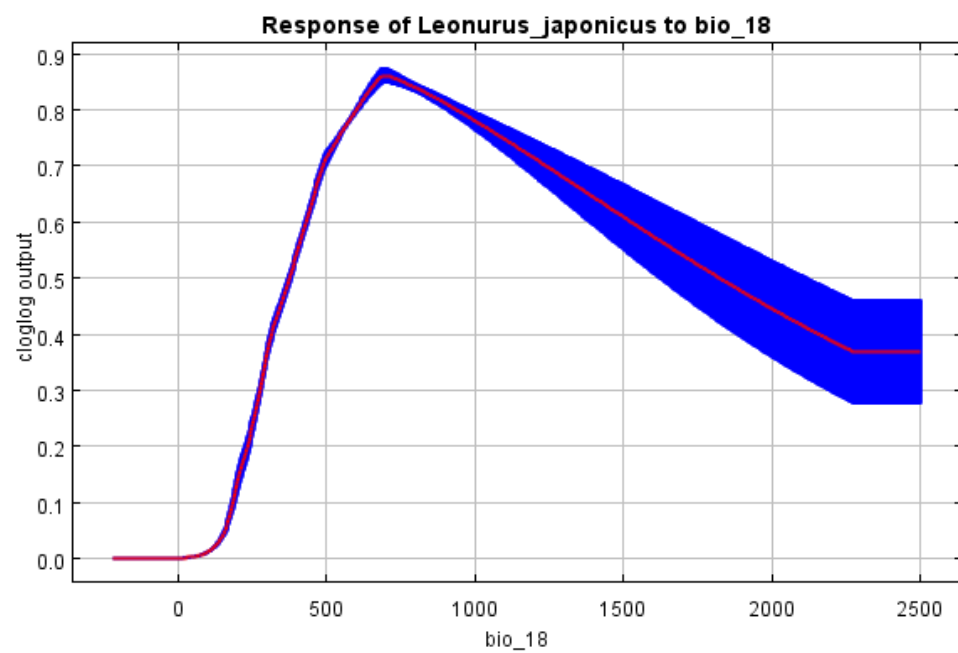

Supplement: Supplementary file 5 — Supplementary Material 5 [file 12870_2023_4284_MOESM5_ESM.pdf]

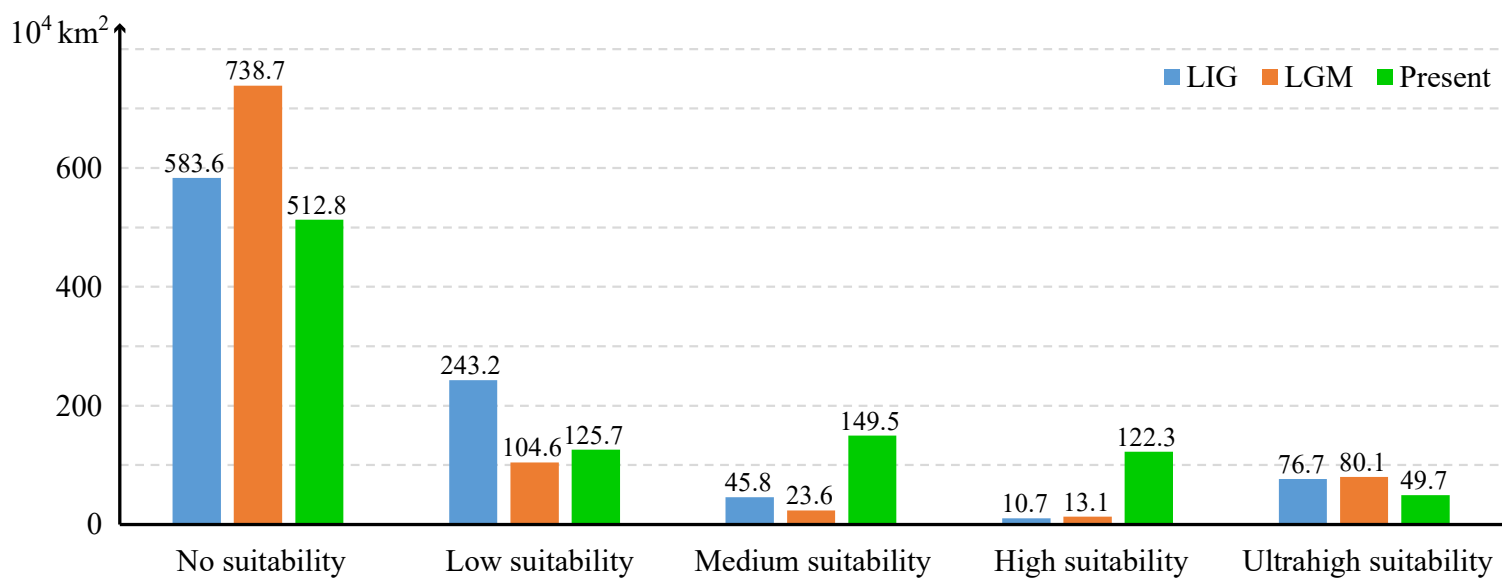

Supplement: Supplementary file 6 — Supplementary Material 6 [file 12870_2023_4284_MOESM6_ESM.pdf]

ML

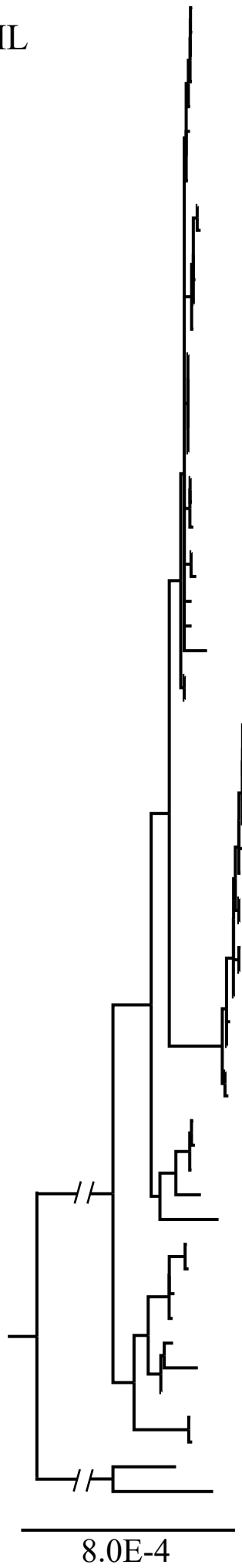

BI

Clade A

Clade B

Clade C

Clade D

Outgroup

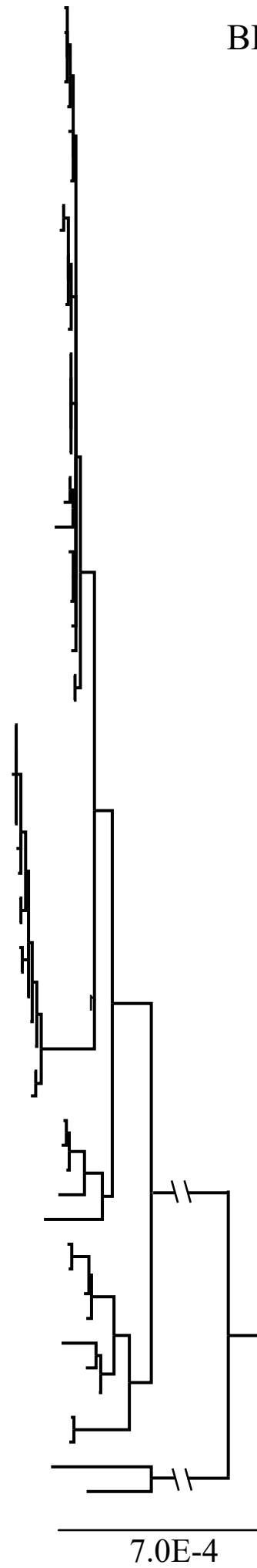

$8.0E-4$

$7.0E-4$

Supplement: Supplementary file 8 — Supplementary Material 8 [file 12870_2023_4284_MOESM8_ESM.pdf]
