# Supplementary material for: Genetic diversity and population divergence of Leonurus japonicus and its distribution dynamic changes from the last interglacial to the present in China
Source: BMC Plant Biol. 2023 May 25;23:276. doi: 10.1186/s12870-023-04284-x (PMC10210291; doi:10.1186/s12870-023-04284-x)
Supplement: Supplementary file 10 — Supplementary Material 10 [file 12870_2023_4284_MOESM10_ESM.pdf]

Supplementary information

Additional file 1: Figure S1. The Pearson correlation analysis among 19 climatic variables using the R package ggpairs.

Additional file 2: Figure S2. The delta K value of STRUCTURE analysis.

Additional file 3: Figure S3. The response curve of the three most dominant climatic variables generated by the MaxEnt model.

Additional file 4: Figure S4. Dynamic changes in the distribution of *L. japonicus* at different suitability levels.

Additional file 5: Figure S5. Phylogram type of Figure1.

Additional file 6: Table S1. Detailed information on the 59 *L. japonicus* accessions in this study.

Additional file 7: Table S2. Detailed information on the GenBank sequences involved in this study.

Additional file 8: Table S3. The description and contribution weight of nine selected climatic variables.

Additional file 9: 49 haplotypes for network analysis.nex
